# Bursting thalamic responses in awake monkey contribute to visual detection and are modulated by corticofugal feedback

**DOI:** 10.3389/fnbeh.2014.00198

**Published:** 2014-05-30

**Authors:** Tania Ortuño, Kenneth L. Grieve, Ricardo Cao, Javier Cudeiro, Casto Rivadulla

**Affiliations:** ^1^Neuroscience and Motor Control Group, NEUROcom, Department of Medicine, University of A CoruñaCoruña, Spain; ^2^Biomedical Research Institute of A Coruna (INIBIC)Coruña, Spain; ^3^Faculty of Life Science, University of ManchesterManchester, UK; ^4^MODES Group, Department of Mathematics, Faculty of Computer Science, University of A CoruñaCoruña, Spain

**Keywords:** attention, burst, LGN, thalamo-cortical, visual

## Abstract

The lateral geniculate nucleus is the gateway for visual information en route to the visual cortex. Neural activity is characterized by the existence of two firing modes: burst and tonic. Originally associated with sleep, bursts have now been postulated to be a part of the normal visual response, structured to increase the probability of cortical activation, able to act as a “wake-up” call to the cortex. We investigated a potential role for burst in the detection of novel stimuli by recording neuronal activity in the lateral geniculate nucleus (LGN) of behaving monkeys during a visual detection task. Our results show that bursts are often the neuron’s first response, and are more numerous in the response to attended target stimuli than to unattended distractor stimuli. Bursts are indicators of the task novelty, as repetition decreased bursting. Because the primary visual cortex is the major modulatory input to the LGN, we compared the results obtained in control conditions with those observed when cortical activity was reduced by TMS. This cortical deactivation reduced visual response related bursting by 90%. These results highlight a novel role for the thalamus, able to code higher order image attributes as important as novelty early in the thalamo-cortical conversation.

## Introduction

Thalamic relay neurons can fire action potentials in two different patterns: tonic mode, in which firing is directly related to the intensity of the visual stimulus, or burst firing. Bursts comprise a number of closely spaced action potentials, induced and coupled by an underlying calcium action potential which is in turn due to the activation of a low threshold (LT) calcium current, which requires a preceding period of quiescent hyperpolarization (Jahnsen and Llinás, 1984a,b; Lu et al., 1992). Hence, these stereotypical bursts represent a non-linear mode of transmission, as the number of action potentials induced appears only weakly related to the stimulus characteristics (Guido et al., 1992), while tonic mode results in firing frequencies more closely related to stimulus attributes. Bursts are usually associated with sleep states (Livingstone and Hubel, 1981; Steriade et al., 1993), but have now been shown to be present in the wake state (Swadlow and Gusev, 2001; Bezdudnaya et al., 2006) thereby suggesting a role as an effective reboot function to the previously quiescent thalamocortical communication channel: the “wake up call” (Guido and Weyand, 1995; Ramcharan et al., 2000; Sherman, 2001a,b; Weyand et al., 2001). This reboot could then itself also result in thalamic changes, perhaps shifting to a tonic mode response, through the large corticofugal feedback pathway which is known to be larger than the actual retinal input to the lateral geniculate nucleus (LGN), directly engaging thalamic relay cells through metabotropic glutamatergic receptors (Godwin et al., 1996).

Paradoxically, another function of this large feedback from cortex to thalamus seems to be to enhance local inhibition, which could, in turn, promote burst firing, as a response to a cortically induced attentional state. Thus the cortex seems to be in the intriguing position, where necessary, of establishing its own wake-up call. Historically, the notion that cortex could use the thalamus to make it attend to interesting stimuli had been suggested earlier by Crick (1984), who then called this the “searchlight hypothesis”, though the nature of the corticothalamic searchlight (or lights) was not made clear. However, though such a model may look attractive, the evidence to date is mixed, with the demonstration of burst firing in the awake state being countered by suggestions that the actual number of such bursts is too low to be relevant (Steriade, 2001; Ruiz et al., 2006). It is clear that attention is a critical factor in this debate.

Here, we have used an attention-based task in the awake monkey to examine the effect of stimulus novelty on the extracellularly recorded visual responses of cells in the LGN. We asked if the presence of a novel stimulus might, through a top-down corticothalamic influence, alter the dynamics of the LGN spiking response, effectively controlling the power of the thalamic drive to the cortex “on-line” during the visual response to the stimulus. Our results show that bursts are concentrated at the initial part of the visual response, and are more numerous in the response to attended target stimuli than to unattended distractor stimuli. Bursts are indicators of the novelty of the stimulus, as task repetition decreased bursting. Furthermore, the primary visual cortex seems to play a relevant role here, modulating burst firing.

## Material and methods

All experiments followed the guidelines of the International Council for Laboratory Animal Science and the European Union (statute nr 86/809) and the protocols were approved by the University of Coruña Committee on Animal Care (CE-UDC30/1/09).

Two male rhesus monkeys (*Macaca mulatta*) were trained to fixate a small spot (0.1°) presented on a video monitor placed at a distance of 57 cm from the animal. Given that the stimuli we used were static flashes involving the form-vision pathway, we have elected to investigate the responses of parvocellular cells. Visual stimulus presentation, single-unit recording and control of behaving trials were carried out using the Cortex software package (NIH). Offline analyses were carried out using custom Spike2 and Matlab routines. Monkeys had to maintain fixation within a 0.6 degree window. Eye position was continuously monitored using an eye tracking system (EyeLink II; SR Research). After 250 ms of maintained fixation, four geometrical figures (three identical and one different) appeared at different positions on the monitor (19″ Belinea CRT monitor, 100 Hz refresh rate). Figures comprised square, circle, rectangle, pentagon, etc. pairs of which were selected randomly for each cell. The luminance of each of the figures was identical and stimulus size was selected so that it covered the receptive field (RF) of the recorded cell, and novel and distractor had the same overall area. All the stimuli were used as target or distractor at different times. Stimuli were presented on a gray background (17.9 cd/m^2^), with the correct polarity, white or black, for the recorded cell (ON or OFF center).

One of the four stimuli was placed over the receptive field of the cell under test, and the others equidistant from the fixation spot, forming a square array. Task 1 comprised detection of the different stimulus (target) while ignoring the distractors. The four figures and the fixation spot remained visible for a randomly selected, variable time of between 1700 and 2000 ms, after which the animal had to make a saccade to the remembered location of the target in order to receive a reward (a drop of juice or water). The trial was automatically aborted if fixation was broken at any time in the task or if the animals signaled an incorrect position. In task 2 the monkey held fixation for 250 ms, at which time a single stimulus appeared at one of the four different positions (arranged as in task 1) and after 500 ms both the figure and the fixation spot disappeared and the animal had again to signal the position of the figure with a saccade; once more, one of the possible positions covered the RF of the recorded cell. For each recorded cell there was one figure that was repeated (distractor) and a small set of others (novel) that appeared randomly. The frequency of appearance of the novel stimulus was on average 1/10. Visual responses were uncontaminated by saccadic eye movements since these occurred only after the visual stimulus disappeared.

Extracellular recording were made in the LGN (Ramcharan et al., 2003), using tungsten electrodes (FHC, Inc.) of 9–12 Mohm resistance. A recording chamber was centered 5.5 mm anterior to interaural zero and 12 mm lateral to the midline. Dura mater was maintained intact during the experiment, and a guide tube was used to place the electrode into the brain; the guide tube remained well above the LGN. LGN recordings were verified by the nature of the visual response and the alternation of the ocular input of the responses as the electrode progressed through the LGN layers (Ramcharan et al., 2003).

To locate the RF of the recorded cell we presented stimuli in each quadrant of the screen. In the quadrant with a response, we repeated the operation, dividing this region into four, and so on, six times, allowing us to define the RF with a precision greater than 1 degree. The actual size of the figures was also adapted to be sure that it fully covered the RF center. Recordings were sorted offline with Spike2 (CED, UK). Spikes were classified as in a burst according to previously established criteria (Guido et al., 1992; Lu et al., 1992): a minimum of two spikes with an interspike interval less than 4 ms. The first spike must have been preceded by a period of silence of at least 50 ms. Thus, the set of all spikes that met these criteria we refer to as “spikes in bursts”. All other activity was considered to be part of the tonic response. This method for detecting LT bursts in extracellular recordings has been used previously with a high degree of certainty (Guido et al., 1992; Lu et al., 1992; Guido and Weyand, 1995; Weyand et al., 2001; Rivadulla et al., 2003; Alitto et al., 2005; Grieve et al., 2009). Trials were aligned with the stimulus onset and analyzed in terms of numbers, distribution, etc. based on which stimulus (target or distractor) was on the RF. Statistical significance was considered when *p* < 0.05.

The probability density functions of spike times were estimated using the kernel method and the Kolmogorov-Smirnov two-sample test was used for detecting possible differences between the target response and the distractor (Rosenblatt, 1956; Parzen, 1962). Data are presented as averaged spikes/trial to allow a comparison of the results independent of the number of trials for each condition.

Principal component analysis (PCA) was carried out on the main characteristics of the bursts (e.g., duration, frequency and inter-burst interval). Cluster analyses were performed separately for target and distractor responses using these characteristics. A Ward method combined with the squared Euclidean distance was used for the cluster analysis. Finally, an analysis of variance (ANOVA) was performed to investigate the influence of the factors cell and trial on the burst characteristics.

Decreased cortical input was achieved using transcranial magnetic stimulation (TMS) over the appropriate part of V1, using a protocol previously demonstrated to induce cortical suppression: repetitive stimulation for 5 min at low frequency (0.8 Hz) (Maccabee et al., 1991; Gangitano et al., 2002). We analyzed the effect of this cortical suppression on components of a visual response (separated as above into tonic firing and bursts) which was tested immediately after TMS application, not during the TMS protocol. In previous control experiments performed in anesthetized cats (for general methods, see de Labra et al., 2007), we applied TMS on V1 with these parameters, recording the visual response of V1 neurons immediately afterwards. In this cat experiment, the electrodes recording cortical activity were cemented in place (tip located approximately in layer VI) prior to positioning of the TMS coil. The results obtained showed that TMS significantly reduced cortical neuronal firing (for an example, see Figure 6, inset).

**Figure 1:**
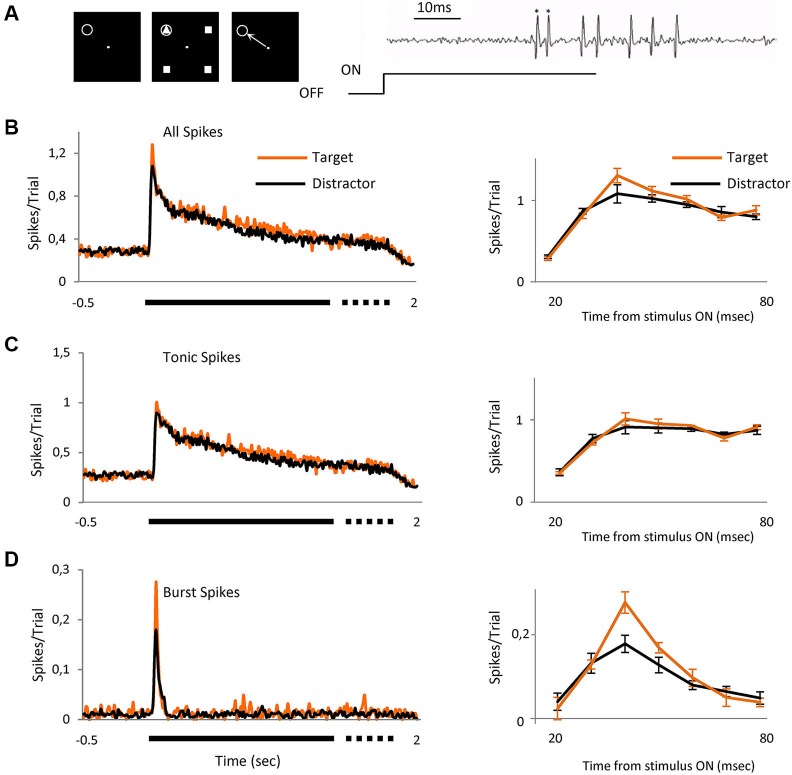
**Bursts signal novel stimulus**. Averaged visual responses of the population of 42 cells. **(A)** Left three panels schematically represent the task. The animal had to fixate a dot in the center of the screen and maintain fixation in a 0.6 × 0.6° window (left panel). Four geometrical figures, (three equal (distractors) and one different (target)) appeared after central fixation (central panel). Stimuli were maintained for a variable time of 1700 to 2000 ms after which the stimuli disappeared and the animal had to signal the location of target with a saccade to that location, which could be over the RF of the recorded cell or in any of the other three positions (right panel). The gray circle indicates the location of the receptive field and was not displayed. Right image shows an example extracellular recording showing a burst (spikes marked *) and tonic spikes (unmarked) mixed within the same recording. **(B)** The left peristimulus time histogram shows the averaged responses of 42 cells to presentation of the two classes of visual stimulus, right PSTH shows a detailed view of the initial part of the response (20 to 80 ms) where the differences between target and distractors are evident (mean ± SEM). **(C)** Represents only tonic spikes and **(D)** spikes in burst. Spikes/trial refers to the average number of spikes for all the trials; it allows a quick comparison between figures, independently of the number of trials. Bin size = 10 ms.

**Figure 2:**
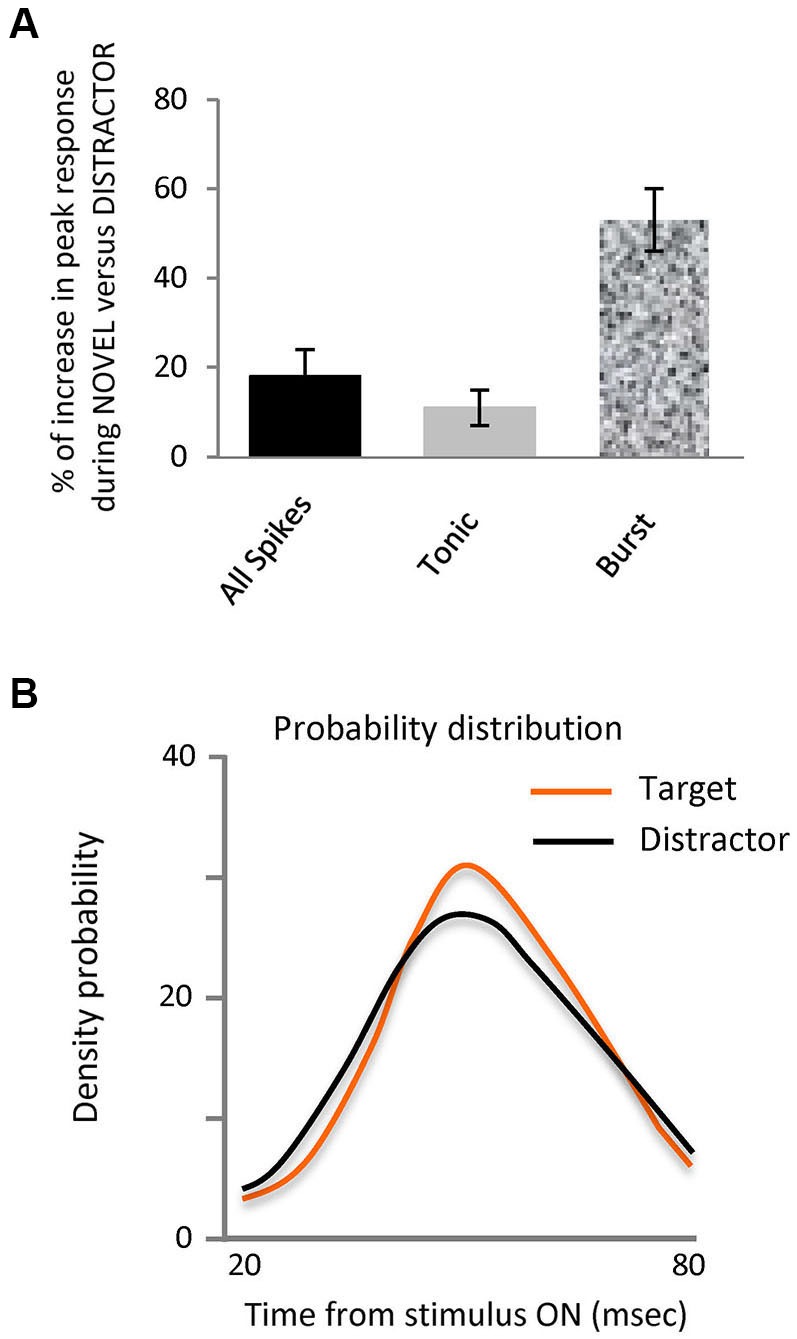
**Novel stimuli evoke more spikes in bursts and are differently organized.** **(A)** Bar histogram representing the increase in peak response to the target stimulus vs. distractors for all spikes, tonic and burst. **(B)** Probability density distribution of spikes in bursts during interval from 20 to 80 ms after stimulus onset for each stimulus, target and distractors. This shows that the distribution of the spikes in bursts within the 20 to 80 ms interval evoked by target falling on the RF was significantly different to those evoked by the distractors, (Kolmogorov-Smirnov Test, *p* < 0.05).

**Figure 3:**
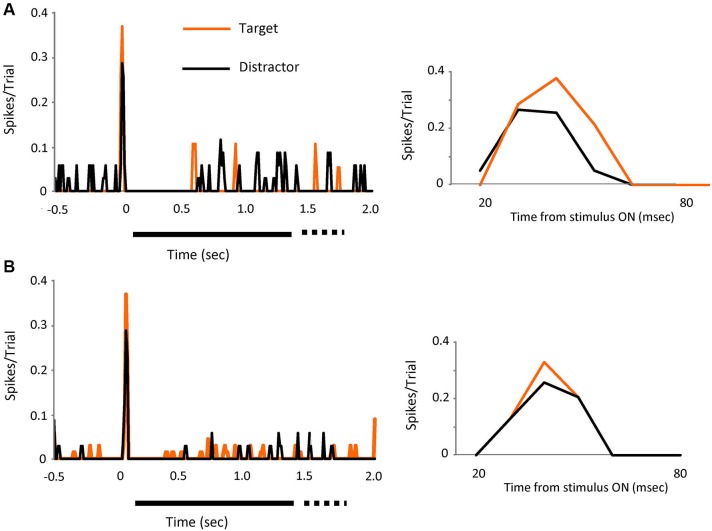
**The effect is visible on individual cells**. Two individual cell examples (**A** and **B**) showing the spikes in bursts (tonic spikes removed) evoked by visual presentation of the target (orange line) or the distractors (black line). Left PSTH’s show increases in peak response for both cells when the target was on the receptive field. Bin size 10 ms. Right PSTH’s shows a detailed view of the initial part of the response (20 to 80 ms).

**Figure 4:**
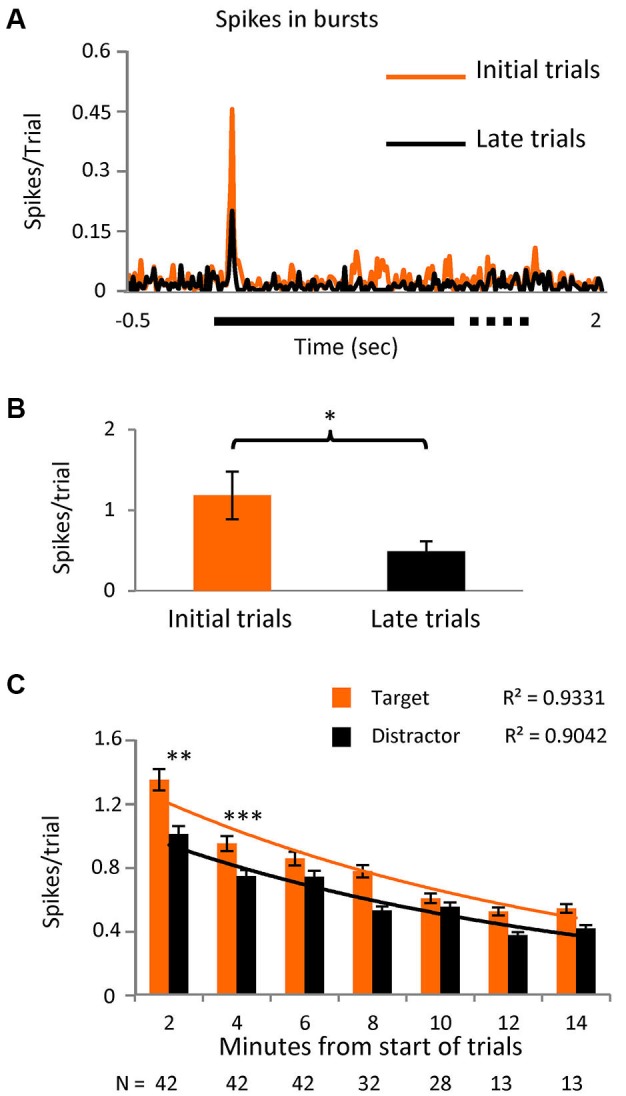
**Initial stimuli evoke more burst responses.** **(A)** PSTH showing the spikes in bursts in the initial part of the task (first third of the trials) and for the later part (last third) in a subset of cells (*n* = 12) where the number of trials was greater than 40 (*p* < 0.05). Bin size = 10 ms. **(B)** Averaged number of spikes in bursts during the initial 20 to 80 ms interval after stimulus presentation in the same subgroup. **(C)** Spikes in bursts with respect to time since the onset of the task, shown as spikes/trial ± SEM and separated into responses to the novel and distractor stimuli. Note that most cells were only tested over 8 min (values below the columns) as outlined in the text describing Figure 3A. An exponential decay curve is shown for both the novel stimuli and the distractor, with the goodness-of-fit values (*r*^2^) given above the traces. The differences between novel and distractor responses were significantly different for the 0–2 and 2–4 min groups (Kolmogorov-Smirnoff test; *p* < 0.005 and *p* < 0.0005 respectively).

**Figure 5:**
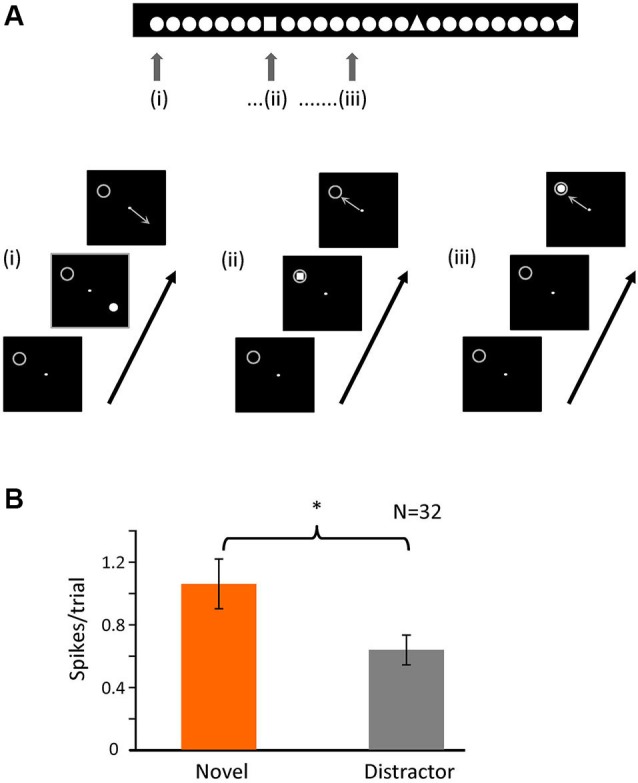
**Novelty of the stimulus is important for bursting**. **(A)** Schematic representation of the second task. The animal had to fixate a dot in the center of the screen and maintain fixation in a 0.6 × 0.6° window. A single figure appeared in one of four different positions of the screen (at the same relative locations as task 1); the image disappeared after 500 ms, and the animal had to indicate the position of the stimulus with a saccade following fixation offset. In each recording session there was one figure that was repeated (distractor) and a small set of others (novel) that appeared randomly within the train as represented by the image above. The gray circle indicates the location of the receptive field and was not displayed. **(B)** Averaged number of spikes in bursts for the initial 20 to 80 ms after stimulus onset when the stimulus (novel or distractor) was over the RF (*p* < 0.05).

**Figure 6:**
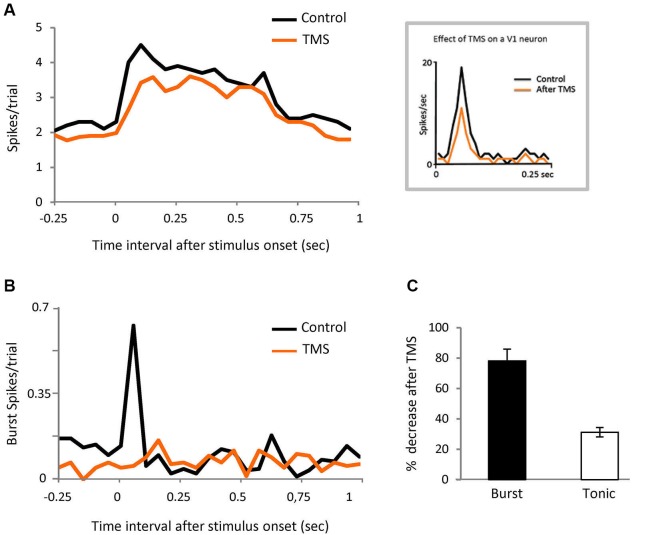
**Corticofugal feedback modulates burst responses.** **(A)** PSTH representing visual responses of the population of LGN cells in control and after TMS in visual cortex (orange). Bin size = 50 ms. Inset shows the response of a V1 neuron recorded in anesthetized cat, in both conditions. **(B)** PSTH showing the effect of TMS on spikes in burst for the same cells shown in panel **(A).** Bin size = 50 ms. **(C)** Comparison of the effect of TMS on spikes in burst and tonic spikes in the initial part of the response (20 to 80 ms).

## Results

### Target stimulus evokes more spikes in bursts

We report here the responses of 82 parvocellular cells recorded from two monkeys, two other cells did not fire spikes in bursts and were discarded from the study. As illustrated in Figure 1A, we arranged the four achromatic stimuli at equal distances around the fixation point on the display such that one fell over the RF of the cell under test. Three of the four stimuli were identical (distractor) and the fourth different (target), shown such that on 25% of trials, randomly interleaved, the target stimulus appeared in the RF and on the other 75% of trials one of the distractors appeared. For each neuron, the target and distractor stimuli were randomly selected from a pool of 12 shapes before each set of trials. Once selected, the target and the distractor remained constant for that cell. The size of the figure completely covered the RF center and was of the appropriate polarity for the cell type, on or off center. The task for the animals (Figure 1A, left panels) was to saccade to the location of the target stimulus in whichever location it appeared, after the stimulus was switched off, for which they were rewarded with water or juice. Figure 1B left shows the averaged responses for the 42 cells recorded during this task, separated into trials in which on the RF was the target (*n* = 993) or a distractor (*n* = 2618). It is already clear from this figure that the initial part of the response to target contains more spikes than distractor, with the peak response 18% higher. The right peri stimulus time histogram (PSTH) shows the response in the initial 20 to 80 ms after stimulus onset, the region of most activity and highest difference. To evaluate the role that the two classes of thalamic responses play in this attentional effect, we separated the entire set of spikes for this period into tonic (Figure 1C) and burst (Figure 1D) and compared the response to the target and distractor. Separation of the response spike trains into burst or tonic components was on the basis of temporal properties (see experimental procedures and Figure 1A, right). The overall percentage of spikes in bursts in this population was 5.31%, in keeping with Ramcharan et al. (2000) who observed 6.6% in their population, using the same criteria we used here. While this appears to be a relatively low proportion, bursting is not homogeneous, spikes in bursts represented 12.5% of all of the spikes in the 20 to 80 ms interval after stimulus presentation. The data was analyzed both by determining the proportion of the overall response which was delivered in bursts, and its temporal distribution, etc. The number of spikes in each burst was on average 2.5 ± 0.056. Statistical analysis did not reveal differences in the observed results between on and off cells; hence results from the total population of cells have been pooled.

Our principal finding is illustrated in Figures 1B–D and 2, showing that the observed differences between target and distractor are attributable to an increased fraction of target responses in burst mode (Figure 1D); whose peak response increased by 53% (Figure 2A, *p* < 0.01 Kolmogorov-Smirnov). The increase in tonic spikes was only 11% and did not reach statistically significance (*p* > 0.05 Kolmogorov-Smirnov). Not only did the overall number of spikes in bursts increase but also the probability of evoking a burst was significantly increased during the responses to the target stimulus, from 10% (274 out of 2618) in the distractor condition to 17% (168 out of 993) during the target presentation (Chi squared; *p* < 0.01).

The effect was visible also in individual cells, as shown in Figures 3A,B, where the response, in spikes in bursts (tonic removed) for two different cells is represented for distractor and target stimuli. Peak responses increased by 32% (upper) and 28% (lower) during target presentation.

Combining trials for distractor and target which had bursts in the first 20–80 ms from stimulus onset showed that spontaneous activity was significantly reduced in the 500 ms before stimulus vs. those trials that had no bursts (35% lower, Chi squared; *p*-Value < 0.01), suggesting, as expected, a hyperpolarized state in those neurons that fired bursts.

Analysis of the distribution of the spikes in bursts within the 20–80 ms interval evoked by target or distractor falling on the RF showed a significantly different distribution (Kolmogorov-Smirnov test, *p*-value < 0.05), as illustrated in the probability density distribution of spikes in bursts in Figure 2B. This shows that burst responses evoked by the target tend to be concentrated in a narrower time interval than those burst responses evoked by the distractors. Not only are there more spikes in bursts in response to the target, they are more focused temporally within the response.

PCA was used to analyze a range of burst characteristics while a cluster analysis compared bursts in the target with those in the distractor condition (see methods). The principal cluster for target (83% of the bursts) was compared to the principal cluster for distractor (84% of the bursts) which revealed that bursts in the target condition contained, on average, more spikes (2.6 ± 0.07) than those in the distractor condition (2.4 ± 0.04, *p* < 0.006, Kolmogorov Smirnov).

We also compared the target response with each of the distractors individually with an ANOVA to detect differences between groups (*p* < 0.007). A multiple range test identified these significant differences as being between target and each distractor but not between the different distractors, confirming the effect of novelty or difference on the level of bursting. Finally, we reasoned that these data might be explained by a decrease in burst firing appearing equally in all distractor data sets, but appearing only in the trial immediately following target appearing over the RF, perhaps the result of *decreased* attention to that location—analysis of this subset of responses vs. the remaining results of the distractor trials showed no significant difference.

In a subset of 12 cells, we extended the number of trials to >40. Logically, we believed that the presence of any stimulus on the RF in the initial trials would confer some novelty, as all stimuli were “new” for the session. We arbitrarily divided the trials into three time epochs and spikes in bursts were shown to be significantly higher (*p* < 0.01) in the initial third compared to the last (Figures 4A,B), confirming our hypothesis. In the later trials however, the difference between spikes in bursts in response to target vs. distractor no longer reached significance, suggesting that as trials progressed “novelty” in the tasks is decreased and bursting becomes less useful. With this in mind we reanalyzed the original data set, selecting only the initial 20 set of trials for each cell and the level of significance of the difference between bursts in response to target vs. distractor now became even higher (*p* < 0.001). Re-examining spikes in bursts in the whole population during task 1, as a simple function of time, we see a near exponential fall off spikes in bursts, steepest over the first few minutes (Figure 4C), with responses to the novel stimulus, measured as spikes/trial, larger than to the distractor, falling closer together later in time.

### Bursts signal novelty

The original task involved a pop-out discrimination of 1 in 4 symbols. In a second, simpler task, shown in Figure 5A, we altered the paradigm to view novelty “in time”—a single distractor stimulus was repeatedly shown, in one of the four locations randomly selected as above (and the animal made a saccadic eye movement to gain a reward, as before), such that in 25% of times it was over the receptive field, but at random intervals, the stimulus was changed to a single presentation of a novel shape—here again the number of spikes in bursts were significantly higher for the presentation of the novel over the receptive field compared to the repeated distractor (Figure 5B). For this task, we recorded 32 cells, giving a total of 2186 trials (910 for target and 1276 for distractor). The percentage of trials containing bursts in the initial 100 ms after stimulus presentation was 21% (189) for the target and 12% (156) for the distractor. Thus here we clearly show that it is the novelty of the stimulus which increases the firing of spikes in bursts, rather than simple attention to the task. Interestingly, as in task 1, the overall numbers of spikes in bursts in response to repeated presentations of any stimulus were significantly higher during the initial presentations compared to later in the trial.

### Cortical input modulates bursting

Since it is known that corticofugal feedback can modulate the proportion of burst and tonic responses in LGN neurons (Wang et al., 2006) we tested the possibility that the cortex is involved in our results by performing the same experiment illustrated in Figure 1A, in this case during transient disruption of cortical activity by TMS, in eight parvocellular cells. We recorded 372 trials pre-TMS (84 target stimuli, of which 15 contained bursts (18%); 288 distractor trials of which 36 contained bursts (12.5%)). We recorded 320 trial post-TMS.

TMS was applied over V1 at 0.8 Hz for 4 min—the observed effect immediately after TMS was applied was a reduction of thalamic neuronal responses that lasted for 6 min (see experimental procedures). We therefore ran the test during that temporal window. The thalamic visual responses were reduced on average by 20% but, interestingly, the effect was not uniform, with the first 100 ms of the response being the most affected (Figure 6A). The burst/tonic ratio was also significantly affected—during the 20 to 80 ms interval the spikes in bursts were reduced by 78% and tonic spikes by 31% (Figures 6B and C). Analysis of the remaining firing showed that there was now no significant different between burst firing in response to target vs. distractor. In the control feline experiments described above, TMS stimulation using the protocol described gave rise to an average visual suppression of 32% (*n* = 6). Facilitatory effects were never observed.

## Discussion

In primates, the lateral geniculate nucleus is the main recipient of visual information from the retina en route to the cortex. As a part of the sensory thalamus, there is an open debate about the actual role of the LGN in visual processing. Within this discussion two main elements seem especially important: the dual nature of the cell firing pattern, tonic and LT bursting, almost unique to thalamus, and the massive corticofugal input, which while apparently influencing a number of thalamic properties has yet to be properly understood (Sherman, 2001a,b, 2007; Steriade, 2001; Rivadulla et al., 2002; Sherman and Guillery, 2002; Cudeiro and Sillito, 2006; Andolina et al., 2007; de Labra et al., 2007; McAlonan et al., 2008; Jones et al., 2012). Based on the proposed role for a burst as a wake-up call from the thalamus to the cortex, we designed an experiment specifically to test the role of bursting as a detector of new/surprising or attention relevant stimuli, and to evaluate the influence of cortical input on such a role. We reasoned that the wake up should not be a global phenomenon affecting the whole visual scene simultaneously but should be limited to specific aspects of the visual image, resulting from a particular shape being at a particular position in visual space, crucially depending upon the period immediately preceding visual stimulation (essentially the lessened synaptic drive for the preceding 50 ms, see methods). We used previously established criteria (see methods and Lu et al., 1992) that guarantee a reliability >98% in detecting LT bursts. This method is broadly accepted and has been applied in several models including awake and anesthetized animals (Guido et al., 1992; Guido and Weyand, 1995; Reinagel et al., 1999; Rivadulla et al., 2003; Alitto and Usrey, 2005). Since our task included only static stimuli, we have decided, for this study, to concentrate on the parvocellular system. Nevertheless, we do not discount the idea that a similar mechanism could be operating at magnocellular level, also programmed by the cortex. It is worth bearing this in mind for future experiments.

Our data is in agreement with others showing attentional effects at the level of the thalamus in monkey and humans (O’Connor et al., 2002; McAlonan et al., 2008). Here we have shown that there is a significant difference in burst firing within the first 100 ms following the appearance of one of four stimuli within the receptive field, such that there is a higher probability of the cell responding to the target with a burst of spikes. By manipulating the appearance of the target and distractor objects between trial sets, we have shown that it is not the shape of the object itself that is important, but simply that it is different from the others, and that this effect does not arise from a temporally delayed withdrawal of attention following the selected, target stimulus. However, the task involves both a straightforward “pop-out” selection of one-in-four targets along with elements of true novelty, in that the nature of the target to be selected was often changed, although the task remained the same. We also observed that the increase in number of responses consisting of bursts was more obvious in earlier trials, where the degree of attention was likely to be higher. This was also reflected in the time course shown in Figure 4C, where significant differences were seen over the first few minutes, the rates falling off exponentially. In our second paradigm, we presented only one stimulus in each trial, in this case the task was simply to locate it and make a saccade to it (ie spatial selection of one of the four locations), without object selection—here novelty was introduced by changing the shape of the target in a relatively small number of trials, unexpectedly. This too resulted in an increased number of initial responses to be a burst within the first 100 ms of stimulus appearance, when the stimulus was different. Here, the change in response seems more akin to a true surprise effect, due to the appearance of an unexpected stimulus, rather than simple attention. Once more the potency of this effect was greater in the early part of trials, diminishing as trial duration continued (and presumably novelty or surprise decreased). The number of spikes in bursts demonstrated here appears small. Nevertheless, placed in the context of a population of cells responding to the presence of each novel stimulus, such spikes in bursts could provide a significant element in the signaling of novelty, potentially amplified in the network of recipient layer 4 cortical cells whose responses to incoming bursts are known to be enhanced (Usrey et al., 2000; Swadlow and Gusev, 2001). McAlonan et al. have also shown a spatial attentional effect on LGN responses, appearing within the first 100 ms after stimulus presentation (McAlonan et al., 2008). They argued that this initial effect was due to a sub-cortical influence clearly involving the thalamic reticular nucleus (TRN) with later attentional effects reflecting feedback from cortex. Their data suggests that our effect on parvocellular cells could be driven by feedback inhibition initiated by magnocellular cells driving TRN GABAergic cells, in turn feeding back into the LGN. The known short latency of magnocellular cells (Maunsell et al., 1999), and their TRN counterparts (McAlonan et al., 2008), fit this scenario well.

The task used by McAlonan differed significantly from ours, however, in that the location to be attended was openly cued in advance, while we instead, in the repetitive blocked nature of the trials, cued four potential locations and the identity of the “different” stimulus, “setting up” a pop-out operation, with one of the four locations becoming the target only after stimulus onset. Visual pop-out phenomena are usually considered to require cortical input (Knierim and Vanessen, 1992; Lamme, 1995), for a review see Albright and Stoner (2002). It is tempting to speculate that the cortical influence on TRN establishes a network “prepared” to signal the attended location of the different stimulus. Thus, while one element of the attention effect of novelty in our paradigm is in the firing of LGN parvo-cellular cells, whereby burst firing is enhanced ie a local, thalamic effect, the overall effect is somehow regulated by the cortico-thalamic input. Our evidence is derived from our observation that during the TMS disruption of cortical activity, the initial part of the visual response of our LGN cells is the most affected and, specifically, bursting is almost completely abolished.

TMS is a very useful tool, capable of reversibly modifying cortically excitability (increasing or decreasing it) based simply upon stimulation parameters (Di Lazzaro et al., 2011). The protocol employed in these experiments utilizes repetitive TMS at low frequency, which has been convincingly shown to reduce cortical excitability in human experiments (Maccabee et al., 1991; Chen et al., 1997; Boroojerdi et al., 2000; Gangitano et al., 2002; Di Lazzaro et al., 2011). This fits well with our previous experiments on anesthetized cats (de Labra et al., 2007 and Figure 6 inset), where repetitive low frequency stimulation on V1 clearly reduced neuronal firing. However, to the best of our knowledge, there are no previously published studies of TMS in visual cortex of awake monkeys combined with single unit recording (a major technical challenge), and, therefore, here we only have an indirect demonstration of this effect. The temporal extension of the effect also varies with the stimulation parameters. In our case, simply by reviewing the modifications observed at the LGN level, we were able to obtain a 5 min period compatible with a reduction of cortical activity for that period. Another critical parameter to bear in mind when using TMS is the spatial extension of the effect (Pascual-Leone et al., 2002). Even using small coils (as we did) in an attempt to be as focal as possible, the extension of the induced current covers a few square millimetres of the cortical surface, far beyond the very small area that can be obtained by using, for example, iontophoretic methods. While this might represent a problem were we trying to make a precise retinotopic blockade that would allow us to study the effect of cortical feedback selectively within a precise sub-domain within the LGN (see Cudeiro and Sillito, 2006 for a review), this was not our aim and the greater extent of our effect was to our advantage. As for the depth of the effect within the cortex, it has been estimated that at a distance of ~3 mm from the skull, the effect will reach the deepest layers of the cortex (de Labra et al., 2007). Having acknowledged these technical constraints, we suggest that rTMS is a valuable tool to evaluate corticofugal feedback in the brain of our experimental animals.

We suggest that our paradigm has evoked a top-down cortical influence selecting pop-out detection of the target figure from one of a specified set of locations (see also McAlonan et al., 2008). This enhances the inhibitory input to these locations (a precondition for burst firing), which may then be “released” on the distractor sites by the lower level connections between thalamus and the reticular nucleus. This enhanced burst response is seen most prominently in the initial minutes of our task 1, indicating that while the task can still be correctly carried out, its “novelty” has been lost. In fact, in this task, the novelty is a function of the new set of locations and stimuli chosen at the start of the trials, for each cell tested. In our task 2, however, the novelty of the odd stimulus remains throughout the task, as the timing of presentation and the shape of the novel stimulus is unpredictable throughout. Here, only the set of locations and the nature of the distractor are cued. However, the spikes in burst again fall off in time, indicating that it is the novelty of the task, rather than its elements, which is important. Interestingly, a very recent study of the cortico-thalamic influences in primate somatosensation found no evidence of an attentional influence at the thalamic level in a wake behaving animal such as ours (Vázquez et al., 2012). Although their task differs significantly from ours, it remains possible that the two sensory systems operate differently, with very different time constraints on sensory discriminations. Our data is in agreement with results obtained by others in awake cats (Weyand et al., 2001). These researchers showed that the incidence of bursts declined with repeated presentation of the stimulus. However our results are in contradiction with those obtained in the monkey by Ruiz et al. (2006), who did not find a relationship between burst firing and stimulus novelty. The most plausible explanation to account for this discrepancy lies upon the completely different task employed. Although they tested a range of behavioral situations, it is still arguable, as they clearly stated in their paper, that the low percentage of spikes in burst mode they obtained was not related to a putative “novelty or surprise signal” because their monkeys were overtrained in the GO-NOGO and target selection tasks. Importantly, the monkeys knew the nature and location of all of the stimuli in advance, with very few trials providing real surprise/novelty. They suggest that this is why they also used natural scenes that had never been presented before, obtaining, again, the same lack of surprise effect. One possible explanation for this could be that the behavioral relevance of the scenes to the monkey was low, that the presentation time was too long (7 s, inducing indifference, if not boredom). Perhaps it would have been different if the animal had been asked to attend some element of the scene for reward. However, in such a case the responses of single cell, whose RF may not meet the criteria required (see above) might be inadequate, and so the use of a multiple recording site electrode might be more successful.

In summary, our data demonstrate that LT bursts in the visual thalamus carry information about some properties of the stimulus, such as novelty or relevance rather than the visual features, and that visual cortex has the capability to regulate thalamic output to increase the power of the burst firing intrinsic to the visual response to the novel stimulus. To accomplish this, cortical feedback needs to maintain an existing attentionally driven inhibitory state which regulates burst firing. We do not suggest that this is “the mechanism” by which the visual thalamo-cortical system indicates novelty, rather that there is an enhanced likelihood of the visual response to a novel stimulus including a burst of firing, or reflect enhanced inhibition at the location of the novel, attended stimulus; since bursts are more likely, and are temporally different under these conditions, so they are more likely to carry significant information.

## Authors’ contributions

Javier Cudeiro, Casto Rivadulla and Kenneth Grieve designed the study. Tania Ortuño, Casto Rivadulla and Javier Cudeiro performed the experiments. Tania Ortuño and Casto Rivadulla analyzed the data. All authors contributed to the scientific discussion, contributed to writing the manuscript and approved the final version for publication. Javier Cudeiro and Casto Rivadulla supervised the study and have an equal contribution to the paper.

## Conflict of interest statement

The authors declare that the research was conducted in the absence of any commercial or financial relationships that could be construed as a potential conflict of interest.
